# Long-range enhancement for fluorescence and Raman spectroscopy using Ag nanoislands protected with column-structured silica overlayer

**DOI:** 10.1038/s41377-024-01655-3

**Published:** 2024-10-28

**Authors:** Takeo Minamikawa, Reiko Sakaguchi, Yoshinori Harada, Hiroki Tanioka, Sota Inoue, Hideharu Hase, Yasuo Mori, Tetsuro Takamatsu, Yu Yamasaki, Yukihiro Morimoto, Masahiro Kawasaki, Mitsuo Kawasaki

**Affiliations:** 1 Department of Systems Innovation, Graduate School of Engineering Science, Osaka University, Osaka, 560-8531 Japan; 2 Division of Interdisciplinary Researches for Medicine and Photonics, Institute of Post-LED Photonics, Tokushima University, Tokushima, 770-8506 Japan; 3 Department of Pathology and Cell Regulation, Graduate School of Medical Science, Kyoto Prefectural University of Medicine, Kyoto, 602-8566 Japan; 4 PRESTO, Japan Science and Technology Agency (JST), Tokushima, 770-8506 Japan; 5 Institute for Integrated Cell-Material Sciences (iCeMS), Kyoto University, Kyoto, 606-8501 Japan; 6 Department of Synthetic Chemistry and Biological Chemistry, Graduate School of Engineering, Kyoto University, Kyoto, 615-8510 Japan; 7 Department of Medical Photonics, Graduate School of Medical Science, Kyoto Prefectural University of Medicine, Kyoto, 602-8566 Japan; 8 Technology and Engineering Division, Ushio Inc., Hyogo, 671-0224 Japan; 9 The Institute of Science and Industrial Research, Osaka University, Osaka, 567-0047 Japan; 10 Department of Molecular Engineering, Graduate School of Engineering, Kyoto University, Kyoto, 615-8510 Japan

**Keywords:** Optical spectroscopy, Biophotonics, Nanophotonics and plasmonics

## Abstract

We demonstrate long-range enhancement of fluorescence and Raman scattering using a dense random array of Ag nanoislands (AgNIs) coated with column-structured silica (CSS) overlayer of over 100 nm thickness, namely, remote plasmonic-like enhancement (RPE). The CSS layer provides physical and chemical protection, reducing the impact between analyte molecules and metal nanostructures. RPE plates are fabricated with high productivity using sputtering and chemical immersion in gold(I)/halide solution. The RPE plate significantly enhances Raman scattering and fluorescence, even without proximity between analyte molecules and metal nanostructures. The maximum enhancement factors are 10^7^-fold for Raman scattering and 10^2^-fold for fluorescence. RPE is successfully applied to enhance fluorescence biosensing of intracellular signalling dynamics in HeLa cells and Raman histological imaging of oesophagus tissues. Our findings present an interesting deviation from the conventional near-field enhancement theory, as they cannot be readily explained within its framework. However, based on the phenomenological aspects we have demonstrated, the observed enhancement is likely associated with the remote resonant coupling between the localised surface plasmon of AgNIs and the molecular transition dipole of the analyte, facilitated through the CSS structure. Although further investigation is warranted to fully understand the underlying mechanisms, the RPE plate offers practical advantages, such as high productivity and biocompatibility, making it a valuable tool for biosensing and biomolecular analysis in chemistry, biology, and medicine. We anticipate that RPE will advance as a versatile analytical tool for enhanced biosensing using Raman and fluorescence analysis in various biological contexts.

## Abstract (web summary)

Remote plasmonic-like enhancement of fluorescence and Raman signals with a > 100 nm silica layer, separating Ag nanoislands from analyte molecules.

## Graphical abstract

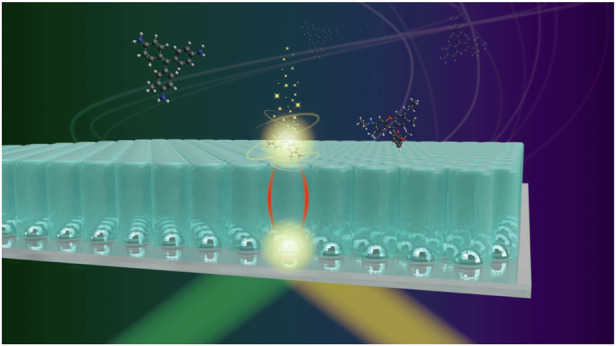

## Introduction

Fluorescence and Raman spectroscopy are essential analytical tools in biology and medicine^1–8^. Fluorescence spectroscopy employs various probes to provide insights into intracellular metabolism and signaling mechanisms^9–12^. Raman spectroscopy, with its ability to offer label-free molecular analysis via molecular vibrations of analyte molecules, provides a noninvasive approach for biological and clinical applications^13–20^. However, currently available biophotonic methods face technical limitations. In fluorescence spectroscopy, the use of sensor molecules as additives can disrupt natural cellular metabolism. To mitigate this, low dosages are necessary, leading to a tradeoff between external perturbation and analytical sensitivity. The sensitivity issue can be more significant in Raman spectroscopy due to the weak signal of Raman scattering, requiring intense and prolonged laser irradiation for signal acquisition, which may potentially cause photodamage to specimens.

Plasmon-enhanced fluorescence and Raman spectroscopy provide a promising solution for analytes with inherently weak optical signals, offering enhanced sensitivity in biosensing and biomolecular analysis. Surface-enhanced Raman scattering (SERS) and surface-enhanced fluorescence have many important applications in biosensing and biomolecular analysis^21–35^. However, the application of localised surface plasmon resonance phenomena to bioanalysis has been limited because the nanostructured metal surfaces need to be in close proximity to the analyte molecules, possibly leading to the mutual degradation of both analyte molecules and metal nanostructures^36–38^. Several researchers have explored modifying the plasmonic nanostructures to extend the effective enhancement range of SERS and surface-enhanced fluorescence^39–41^. Nevertheless, achieving long-range enhancement with a sufficient enhancement for bioanalysis remains a challenge, given the fundamental enhancement mechanism of SERS and surface-enhanced fluorescence.

In this paper, we report long-range fluorescence and Raman scattering enhancement via dielectric nanostructures of over 100 nm thickness, namely, remote plasmon-like enhancement (RPE). The key element enabling RPE is the column-structured silica (CSS) layer, which protects the mutual adverse impact between a dense random array of Ag nanoislands (AgNIs) and analyte molecules. The experimental evidence presented in this study supports the concept of RPE in terms of its phenomenological aspects. Additionally, we have successfully demonstrated the practical application of RPE in enhancing fluorescence biosensing for live cells and Raman imaging of biological tissues.

## Results

### Key structural elements of RPE plate

**Fig. 1:**
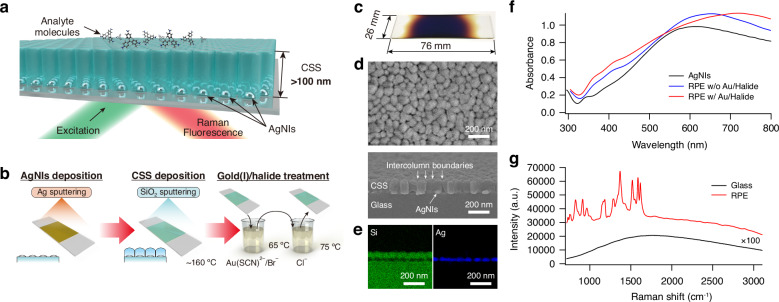
Dense random array of AgNIs coated with CSS overlayer of over 100 nm thickness for RPE. **a** Schematic diagram of an RPE plate. Analyte molecules are positioned on top of the CSS structure, where the CSS layer acts as a separation layer between the AgNIs and the analyte molecules. **b** Schematic diagram illustrating the fabrication process for an RPE plate. **c** A photograph of an RPE plate. The brownish area indicates where the AgNIs and the CSS overlayer are constructed. **d** FE-SEM images displaying the top view and oblique cross-section of an RPE plate, revealing a 120 nm thick CSS layer covering the AgNIs. **e** Energy-dispersive X-ray spectroscopy analyses of RPE plates. The AgNIs are between the slide glass basal bottom and CSS upper layers. **f** Extinction spectra of the AgNI and RPE plates, including RPE plates with and without gold(I)/halide bath treatment. **g** Spectral comparison of FUC molecules embedded in PVA on RPE and glass plates. PVA-embedded FUC was spin-coated on the RPE and the glass plates with a thickness of 200 nm containing 3 × 10^12^ molecules/cm^2^. The emission spectrum using the glass plate was scaled 100 times for visibility

A schematic diagram of the RPE plate is shown in Fig. 1a. The key structural elements of the RPE plate are a dense random array of AgNIs on a slide glass basal plate and a CSS overlayer. These key structures were fabricated by the sputtering process, as shown in Fig. 1b (see Methods for more detail). A dense array of AgNIs was grown by direct-current Ar^+^ ion sputtering onto the smoother side of a float slide glass plate in 5 min. The deposition of a CSS layer on the AgNIs layer was conducted using radio-frequency sputtering with a SiO_2_ deposition rate of 10 nm/min. The high-energy discharge plasma caused the substrate temperature to rise to 160 °C. Since the sputtering process can be employed to construct the key structural elements of both the AgNIs and CSS overlayer, the RPE plate can be efficiently produced on a large scale, as shown in Fig. 1c.

Each AgNI measured 50–150 nm in lateral dimension and approximately 20 nm in height (Fig. S1). Unless otherwise protected, such AgNIs are highly vulnerable that they do not even withstand aerobic corrosion and undergo immediate corrosive dissolution in saline, i.e., under a common aqueous environment for biological experiments. The CSS layer, ~100 nm or more in thickness, works as a robust protection layer for the AgNIs. A cross-sectional field-emission scanning electron microscopy (FE-SEM) image revealed a 120 nm-thick CSS layer with directional intercolumn boundaries, as shown in Fig. 1d. The intercolumn boundaries originated predominantly from the interstitial positions of the respective AgNIs. In the growth of CSS, the structure of AgNI did not change and was localised between the substrate and the CSS coating layer, as shown in Fig. 1e.

After constructing the fundamental structure of the RPE plate, an additional step of gold(I)/halide bath treatment was performed to enhance its functionality. We characterised the extinction spectrum to investigate the fundamental plasmonic properties of the developed RPE plates. The dense arrays of AgNIs without the CSS overlayer exhibited broad plasmon bands that spanned the entire visible region and even extended into the near-infrared region, as shown in Fig. 1f. Introducing the CSS overlayer caused a noticeable red shift of this broad plasmon band. Subsequently, the gold(I)/halide bath treatment led to further significant broadening and red shift of the plasmon band. The observed features are likely attributed to the silver-gold nanoalloying, which alters the optical properties of the AgNIs but also potentially influences the plasmon-plasmon interactions between adjacent nanoislands. It is important to note that during the gold(I)/halide bath treatment, gold is localised near the AgNIs and not deposited on the CSS surface (Fig. S2).

### Fundamental confirmation of RPE

To evaluate the enhancement capability of the RPE plate, we analysed the emission spectrum of basic fuchsin (FUC) and rhodamine 6 G (R6G) molecules on the RPE plate with gold(I)/halide treatment. The RPE plate exhibited a substantial enhancement of the Raman and fluorescence signals of FUC in comparison to the glass plate, as shown in Fig. 1g. The enhancement factor (EF) reached up to 2 × 10^7^ in the Raman spectrum of R6G (Fig. S3) and 170 in the fluorescence spectrum of FUC (Fig. 1g) for the RPE plate with both measurements employing an excitation wavelength of 532 nm. This result suggests that the RPE plate has a sufficient enhancement effect for fluorescence and Raman spectroscopy.

Remarkably, significant enhancement of RPE was observed even in RPE plates without the gold(I)/halide bath treatment; for example, the EF reached an order of 10^6^ in R6G, as shown in Fig. S4. Incorporating the gold(I)/halide bath treatment further amplified the enhancements in Raman scattering and fluorescence by several to tenfold. Notably, when only the plasmonic properties of the AgNIs degraded without the degradation of the CSS, the intensified fluorescence and Raman scattering signals weakened (Fig. S5). Consequently, it becomes apparent that the key elements for achieving RPE are the plasmonically-activated AgNIs and CSS. The gold(I)/halide bath treatment acts as an additional component, effectively enhancing the functionality of RPE.

### Experimental proof of separation of analyte molecules from AgNIs in RPE

To prove that the analyte molecules and AgNIs were indeed separated by a CSS layer with a thickness greater than 100 nm, the location of the analyte molecules was carefully inspected from various viewpoints.

**Fig. 2:**
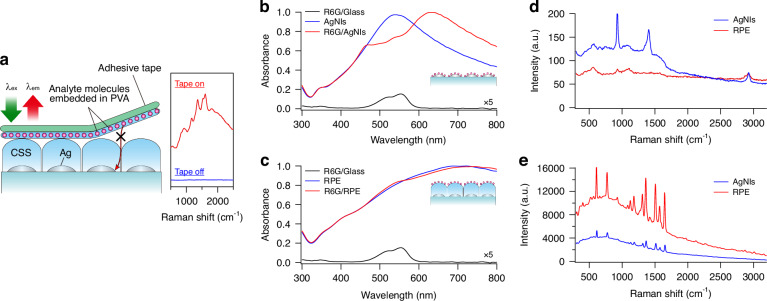
Experimental validation of remote enhancement using RPE plates without close proximity between analyte molecules and metallic nanostructures. **a** Adhesive tape test. Raman spectra were obtained with and without the attachment of adhesive tape containing FUC molecules embedded in PVA on the adhesive side. The adhesive tape was lightly attached to or removed from the CSS surface. The excitation wavelength was 532 nm. **b** Extinction spectra of R6G-spin-coated AgNI plate. Extinction spectra were measured for R6G with 3 × 10^14^ molecules/cm^2^ coverage. Additionally, a reference spectrum was obtained for R6Gs spin-coated onto a glass plate, and it is presented on an expanded (5 times) absorbance scale. **c** Extinction spectra of R6G-spin-coated RPE plate. The observation conditions were identical to those of the AgNI plate. **d** Emission spectra of PVA on AgNI and RPE plates. 5 wt% PVA was dropped onto each plate, and emission spectra were obtained with an excitation wavelength of 532 nm. **e** Emission spectra of PVA-embedded R6G on AgNI and RPE plates. R6G mixed with 5 wt% PVA was dropped onto each plate, and emission spectra were obtained with an excitation wavelength of 532 nm

Firstly, as a preliminary confirmation, we rinsed the spin-coated R6G molecules on the RPE plate with ethanol for a brief few seconds. As a result, the enhanced emission spectrum of R6G was simultaneously extinguished. This observation could only explain if the spin-coated R6G molecules were weakly bound to the surface of the CSS layer. To further verify that the analyte molecules were attached to the top of CSS, we performed an adhesive tape test. We attached FUC molecules embedded in PVA onto the adhesive side of the tape, as illustrated in Fig. 2a. We observed that the Raman signals of FUC were enhanced only when the tape was attached to the CSS surface of the RPE plate. Moreover, these signals appeared and disappeared reversibly upon attachment and removal of the tape from the surface of the CSS. Importantly, we did not detect any Raman signals of FUC when the tape was attached to the glass plate.

Secondly, we verified the precise positioning of analyte molecules based on their interaction with plasmons. Upon applying R6G to an AgNI plate, the R6G molecules would adhere to the AgNIs. This led to a plasmon-molecule near-field coupling that caused significant alterations in the extinction spectrum, as shown in Fig. 2b. In contrast, this change was absent when the R6G molecules were deposited onto the RPE plate, as shown in Fig. 2c. As a side note, the slight distortion observed in the extinction spectra near the absorption wavelength of R6G in R6G-coated RPE plates, as opposed to those without R6G, can be attributed to a classical optical effect arising from a multi-layer model.

Thirdly, we investigated the difference in enhancement properties between the RPE and AgNI plates. The Raman spectrum of polyvinyl alcohol (PVA) was enhanced on the AgNI plate but not on the RPE plate, as shown in Fig. 2d. The AgNI plate can enhance the emission spectrum of PVA through the near-field enhancement effect. This enhancement can be attributed to the close proximity between the PVA molecules and AgNIs. The lack of enhancement in the emission spectrum of PVA on the RPE plate suggests that the PVA molecules did not reach the AgNIs on the RPE plate. However, interestingly, we found that the emission spectrum of certain PVA-embedded molecules, such as R6G, could be significantly enhanced in the RPE plate, with an EF of approximately 2 × 10^7^, similar to or even greater than the enhancement observed in the AgNI plate, as shown in Fig. 2e. This indicates that the observed enhancement in the RPE plate occurred even in the absence of direct contact between the PVA-embedded R6G and the AgNIs. Furthermore, the differences observed between the enhancement of PVA alone and PVA-embedded R6G in the AgNI and RPE plates suggest that the enhancement mechanism involved with the RPE plate differs from the near-field enhancement exhibited by the AgNI plate. These findings indicate a distinct mechanism at play in the RPE plate, and it is evident that the R6G molecules are somehow separated from the AgNIs by a certain distance.

Other compelling evidence supporting the CSS surface-confined localisation of analyte molecules was obtained through X-ray photoelectron spectroscopy (XPS) surface analysis of the CSS layer with spin-coated R6G (Fig. S6). The relative intensity of the N_1s_ signal from R6G, compared to the Si_2p_ signal from the CSS layer, could only be justified if most R6G molecules were located within the XPS analysis depth of less than 2 nm. Furthermore, a brief Ar-ion etching process, which selectively removed thin surface adsorbates, was sufficient to eliminate the N_1s_ signal attributed to R6G. If any unobserved R6G molecules were present in the CSS gap and contributing to enhanced Raman signals, the Raman EF estimated based on the total coverage of R6G would become unreasonably higher (as seen in the signal comparison between the SERS signal in Fig. 2e). These observations conclusively rule out the possibility that the spin-coated R6G penetrated deeply into the CSS layer along the intercolumn boundaries and approached the metal surface at close proximity.

### Unique enhancement characteristics in RPE-enhanced spectroscopy

**Fig. 3:**
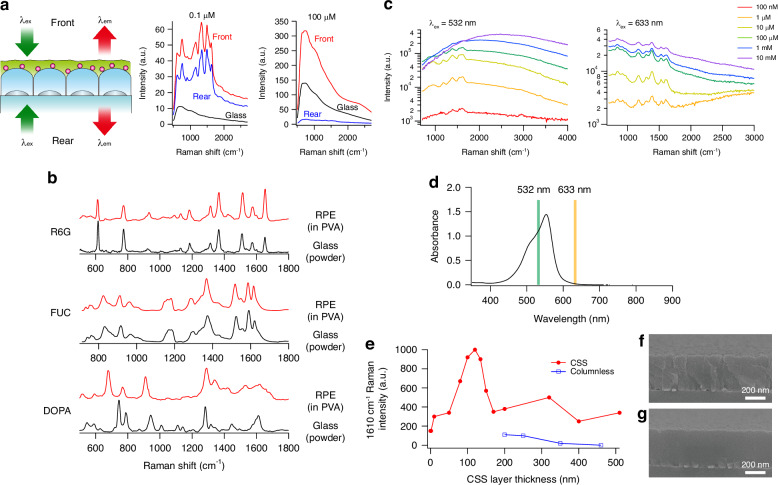
Unique characteristics of RPE-enhanced spectroscopy. **a** RPE-enhanced emission spectra obtained with front- or rear-side excitation and on the same side collection. The spectra show the overlapping Raman scattering and fluorescence signals of R6G at concentrations of 0.1 µM and 100 µM embedded in PVA films, along with a reference spectrum of R6G on a transparent glass plate. **b** Comparison of Raman spectra of R6G, FUC, and dopamine (DOPA) using RPE and glass plates. RPE measurements were conducted on analyte molecules dissolved in PVA, while measurements on glass plates utilised the powdered form of the analyte molecules. **c** Excitation wavelength dependency for RPE observed in FUC dissolved in PVA. RPE-enhanced emission spectra of the FUC concentration series were obtained at wavelengths of 532 nm (rigorous resonance condition) and 633 nm (away from the resonance condition). **d** Extinction spectra of 100 µM FUC dissolved in water. **e** RPE-enhanced Raman intensity of spin-coated R6G as a function of the thicknesses of CSS. The Raman intensity at 1610 cm^−1^ for spin-coated R6G at 633 nm excitation, with varying thicknesses of CSS and columnless silica structures on RPE plates, are shown. **f** Representative FE-SEM image of a 400-nm-thick CSS. **g** Representative FE-SEM image of columnless silica structure on AgNIs

To investigate the enhancement effect of RPE, we first examined the EFs in Raman scattering and fluorescence signals. Initially, we observed a significantly higher EF in Raman scattering compared to fluorescence, as discussed in the previous section and shown in Fig. 1g. This finding suggests that the enhancement mechanisms for Raman scattering and fluorescence in RPE are distinct. Furthermore, we discovered that the EFs in fluorescence varied depending on the molecular spectroscopic properties. For instance, the fluorescence EF of R6G reached a maximum of approximately 4, as shown in Fig. 3a. R6G acts as a fluorophore with a high fluorescence quantum yield (>0.95) both in solution and PVA^42^. Considering its original fluorescence quantum yield, which is close to unity, it can be inferred that the additional enhancement mainly arises from an increase in the excitation efficiency, i.e., the effective absorption cross-section. In contrast, the fluorescence EF of FUC is approximately 20-fold higher than that of R6G, as shown in Fig. 1g. Despite FUC exhibiting a lower fluorescence quantum yield compared to R6G, even in the PVA, these differences in EFs indicate that RPE is not solely responsible for the observed electric field enhancement.

Next, we examined two opposite optical configurations, namely front- or rear-side excitation with emission collected on the same side, as shown in Fig. 3a. Initially, we used 0.1 µM PVA-embedded R6G dispersed on an RPE plate and measured the emission spectra for both optical configurations. Surprisingly, we observed enhanced emission spectra, particularly in the Raman signal, for both configurations, and the signal intensities were similar. However, upon increasing the concentration of R6G embedded in PVA to 100 µM, the Raman bands disappeared, while moderate fluorescence signals remained detectable. At the higher concentration case of 100 µM R6G, the fluorescence intensities are reasonably explained by assuming the AgNI layer is considered a semitransparent mirror with a transmittance of approximately 10-20%. This implies that the excitation and emission light are reflected by the AgNI layer, resulting in a stronger signal in the front-side (sample side) configuration and a weaker signal in the rear-side configuration. In summary, one notable feature of RPE is the absence of excitation direction dependence, which becomes evident at low molecular concentrations.

We also examined the characteristics of the spectral shape of the RPE-enhanced Raman spectra. The enhanced Raman spectra on the RPE plate and spontaneous Raman spectra on the glass plate were compared, as shown in Fig. 3b. Notably, we observed a general consistency between the RPE-enhanced Raman spectra and the spontaneous Raman spectra for R6G and FUC. However, an intriguing observation emerged when analysing molecules such as Dopamine, where the spectral shape exhibited significant deviations from the spontaneous Raman spectra. This result indicates a strong dependency of enhancement properties in RPE on the particular molecular state of the analyte molecules, similar to SERS. However, it is important to note that RPE clearly differs from the enhancement by SERS, as demonstrated in the previous section. It underscores that the interaction between the RPE plate and the analyte molecules involves intricate mechanisms beyond simple electric field enhancement, suggesting the presence of additional contributing factors.

We also investigated the excitation wavelength dependency for RPE. A FUC concentration series of RPE-enhanced emission spectra at excitation wavelengths of 532 and 633 nm are shown in Fig. 3c. At 532 nm, where FUC exhibited rigorous electronic resonance, as shown in Fig. 3d, both Raman and fluorescence intensities increased proportionally at lower concentrations (<10 μM). In contrast, in higher concentrations (>100 μM), nonlinear dependence of the emission spectra on molecular concentration was observed, in which the Raman signal became invisible and the fluorescence signal was saturated. At 633 nm, which is outside the strict resonance condition, Raman enhancement decreased by an order of magnitude compared to the resonant case, but proportional enhancement was kept up to 100 μM. These findings demonstrate that significant Raman and fluorescence enhancement occurs when the excitation wavelength aligns with the electronic resonance wavelength of FUC molecules. Furthermore, signal saturation is observed at lower molecular concentrations under resonance wavelength conditions, while higher concentrations are required when the excitation wavelength deviates from the resonance wavelength. These results suggest a correlation between the RPE phenomenon and the electronic resonance of the analyte molecules.

We also evaluated the dependence of Raman spectrum enhancement on the thickness of the CSS. Surprisingly, we observed a remarkable enhancement of the Raman spectrum of R6G over the CSS thickness range of approximately 0 to 500 nm, reaching the EF of 10^6^ to 10^7^, as shown in Fig. 3e. Notably, at the lower end of the CSS thickness range (0 nm), the structure solely consists of AgNIs, indicative of SERS. Interestingly, this enhancement was only observed in the CSS with clearly distinguishable intercolumn boundaries (Fig. 3f), but not in a virtually columnless silica structure (Fig. 3g). Furthermore, we found that the Raman enhancement exhibited a distinct peak at a CSS layer thickness of 120 nm, which coincided with the position of the local maximum amplitude of the excitation light at 633 nm. This maximum enhancement might be generated by an interference effect involving the dense layer of AgNIs acting as an optical half mirror, located 110 nm from the metal surface for a wavelength of 633 nm and a refractive index of 1.42. We observed the local maxima of the signal enhancement at approximately 330 nm and 550 nm, which coincided with the second- and third-order constructive interferences. However, it is important to emphasise that this interference effect only adds a minor EF to the Raman enhancement, and it is not essential for the observed Raman enhancement, which yields significantly higher EF with an order of 10^6^ to 10^7^. These findings indicate that the RPE occurs with a CSS involving well-defined boundaries, and there exists an optimal CSS height that contributes to this enhancement.

### Practical advantages of RPE-enhanced spectroscopy

**Fig. 4:**
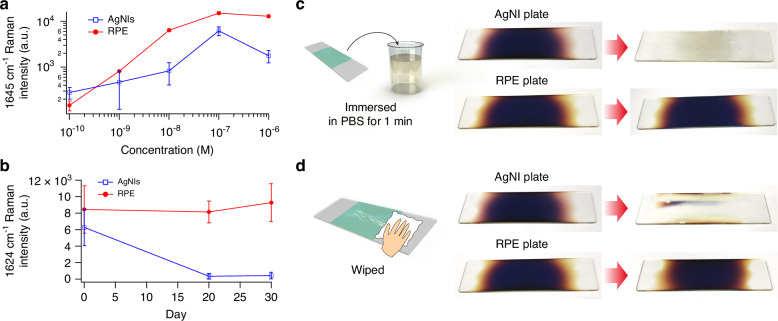
Practical advantages of RPE-enhanced spectroscopy. **a** Molecular concentration dependency for Raman intensity of R6G on RPE and AgNI plates. PVA-embedded R6G was dropped to each plate, and the Raman intensity at 1645 cm^−1^ was measured. The excitation wavelength and power were 532 nm and 0.25 mW, respectively. Mean values ± standard deviation are shown (*N* = 5). **b** Long-term stability of RPE plates. Typical long-term changes in Raman intensity at 1624 cm^−1^ for a 100 µM ethanolic solution of crystal violet on AgNI and RPE plates are shown. The samples were exposed to air at room temperature. Mean values ± standard deviation are shown (*N* = 3). **c** Chemical durability of the RPE plate. AgNI and RPE plates were immersed in PBS at room temperature for 1 min. **d** Physical durability of the RPE plate. AgNI and RPE plates were wiped with a paper towel. The slide glass basal plates measure 26 mm in width and 76 mm in length

The Raman and fluorescence spectroscopy using the developed RPE plate offers several practical advantages. Firstly, we assessed the signal behaviour of RPE-enhanced spectroscopy. The variation in the intensity of a representative Raman band of R6G (1645 cm^−^^1^) with respect to molecular concentration for the rear-side measurement is demonstrated in Fig. 4a. The Raman signals were greatly enhanced through the RPE effect, facilitating measurements at exceedingly low molecular concentrations, extending into the nanomolar range or even lower. The signal-to-noise ratio (SNR) remained high up to the concentration of 10 nM (SNR 16.5 at 10 nM). Note that the saturation was observed above the concentration of 100 nM, which was attributable to the characteristic saturation phenomenon of RPE described in the previous section. In contrast, linearity and SNR were significantly lower when conducting SERS spectroscopy with an AgNI plate under the same conditions as RPE-enhanced spectroscopy (SNR 1.8 at 10 nM). This discrepancy arises from the lack of strict control over the position of analyte molecules in SERS, leading to substantial signal variations depending on whether the molecule resides within a hot spot featuring a short AgNI inter-gap distance. These findings underscore the capability of RPE-enhanced spectroscopy for quantitative molecular sensing, exhibiting both high SNR and linearity. Unlike SERS spectroscopy, RPE-enhanced spectroscopy does not require precise control over the placement of analyte molecules, resulting in consistent and reliable measurements.

Secondly, we evaluated the stability of the RPE plate in production and storage, considering factors such as reproducibility, spatial uniformity, and long-term stability. To assess reproducibility, we employed 10 individual RPE plates that were fabricated separately. A relative standard deviation (RSD) exhibits 14% for Raman scattering and 9% for fluorescence (Fig. S7) when employing PVA-embedded FUC as analyte molecules. In terms of spatial uniformity, the RSD values for Raman scattering and fluorescence are 16% and 14%, respectively (Fig. S8). Moreover, the Raman signal of crystal violet, initially prepared on day 0, exhibits remarkable stability over a minimum period of 30 days when utilising the RPE plate, as shown in Fig. 4b. In stark contrast, the Raman signal using the AgNI plate shows rapid weakening over time. These findings highlight the long-term stability advantage of the RPE plate. Collectively, these results demonstrate that RPE plates offer a practical advantage in terms of stability, exhibiting reproducibility, spatial uniformity, and long-term stability.

Finally, we conducted an evaluation of the chemical and physical durability of the RPE plate. Generally, the silver nanostructure (AgNI) is known to be chemically unstable. When the AgNI plate, with exposed silver nanostructures, was immersed in phosphate-buffered saline (PBS), a commonly used solution in biological experiments, the discolouration was observed within 1 min, as shown in Fig. 4c, indicating its chemical instability. In contrast, when the RPE plate was subjected to the same PBS immersion, no discolouration occurred. This remarkable chemical durability can be attributed to the avoidance of contact between AgNIs and chemicals by the protective CSS layer and the improved chemical resistance of AgNIs by the gold(I)/halide bath treatment. Furthermore, the presence of the protective CSS layer significantly improves the physical durability of the RPE plate, as shown in Fig. 4d. When the surface was wiped with a paper towel, the RPE plate exhibited no changes. On the other hand, the AgNI plate could be easily wiped off due to inadequate adhesion between the AgNIs and the slide glass basal plate. These results demonstrate that the RPE plates possess chemical and physical durability, making them highly valuable for practical applications.

### RPE-enhanced fluorescence spectroscopy for live cells

**Fig. 5:**
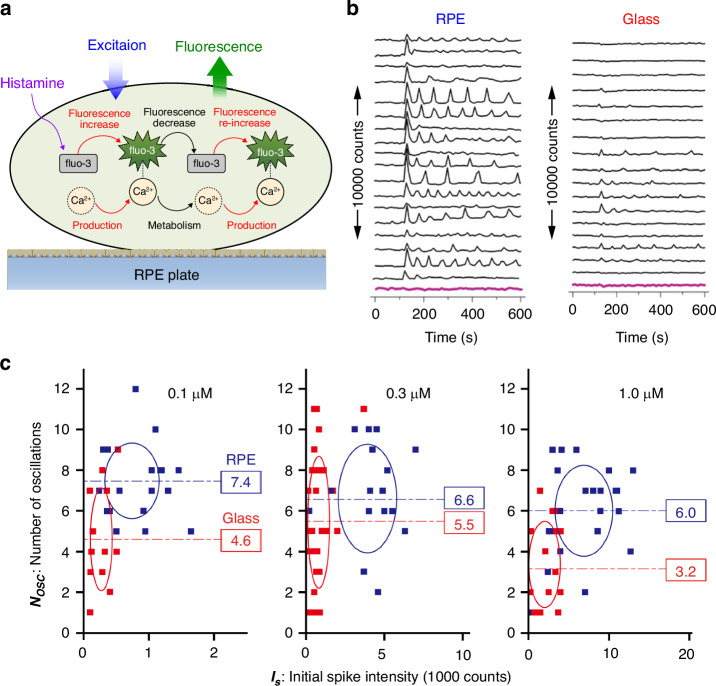
RPE-enhanced fluorescence spectroscopy for histamine-induced Ca^2+^ oscillation in HeLa cells. **a** Schematic representation of Ca^2+^ oscillation triggered by histamine and monitored using fluo3-AM as a Ca^2+^ biosensor. **b** Temporal fluorescence signal profile indicating the levels of Ca^2+^ in HeLa cells cultured on RPE or glass plates. Ca^2+^ oscillation was induced by 1 µM histamine, with fluo3-AM loaded at a concentration of 0.1 μM in the extracellular medium. The pink lines represent the instrumental noise level. **c** Scatter plots demonstrating the initial fluorescence spike intensity (*I*_*s*_) and the number of oscillations (*N*_*osc*_) for three different levels of fluo3-AM loading. The time window for counting *N*_*osc*_ is defined as 480 s starting from the initial spike of Ca^2+^ oscillation. The numbers within the boxes indicate the average *N*_*osc*_ values. The centre of the ellipses corresponds to the mean of *I*_*s*_ and *N*_*osc*_, while the respective axial diameters correspond to twice the standard deviations of *I*_*s*_ and *N*_*osc*_, respectively

We evaluated the enhanced fluorescence detection capacity of RPE in the application of fluorescence biosensing. Specifically, we investigated the RPE-enhanced fluorescence detection of Ca^2+^ oscillation in live HeLa cells. To promote healthier cell growth, we precoated the RPE plate with Matrigel, a protein that facilitated cell adhesion and growth. The effectiveness of Matrigel coating on the RPE plate was confirmed by comparing the growth of HeLa cells on the RPE plate with that on a standard Matrigel-coated glass plate (Fig. S9). For the Ca^2+^ sensing, we utilised fluo3-AM dyes, a widely used cytosolic Ca^2+^ sensor in biosensing technology^43^. The oscillatory dynamics of Ca^2+^ production and metabolism were assessed through the fluorescence intensity of fluo3-AM dyes, as illustrated in Fig. 5a. Fluorescence excitation for fluo3-AM was conducted using a wide-field fluorescence microscope with an excitation wavelength of 480 nm.

The time response curves of Ca^2+^ levels of several cells induced by histamine stimulus at the fluo3-AM loading level of 0.1 μM in the extracellular medium are shown in Fig. 5b. Notably, the fluorescence signal of HeLa cells cultured on the RPE plate exhibited significant enhancement compared to that on the glass plate. This enhancement facilitated the clear visualisation of Ca^2+^ oscillation in HeLa cells induced by histamine.

To further elucidate the enhancement effect of RPE, we employed two quantitative measures from the time response curve: the initial fluorescence spike intensity (*I*_*s*_) triggered by the histamine stimulus and the number of Ca^2+^ signal oscillations (*N*_*osc*_). *I*_*s*_ represents the initial transient rise in the concentration of the complex between fluo3-AM and Ca^2+^ within each cell, while *N*_*osc*_ provides a statistical measure of the intrinsic signalling dynamics of Ca^2+^. The distributions of *I*_*s*_ and *N*_*osc*_ are presented in Fig. 5c. For our analysis, we focused on HeLa cells with *I*_*s*_ values above the noise level. Notably, as the fluorescence intensity of *I*_*s*_ was strengthened by RPE, *N*_*osc*_ also tended to increase. This observation suggests that the enhanced Ca^2+^ oscillation signal on the RPE plate allowed for better detection, whereas, on the glass plate, the oscillation signal would be obscured by noise. These results demonstrate the feasibility and efficacy of RPE in fluorescence biosensing for live cells and serve as proof of principle for its application in this field.

### RPE-enhanced Raman spectroscopy for tissue imaging

We show next how RPE works in histological Raman imaging. A tissue section of the oesophagus with the oesophagal adventitia from a Wistar rat was affixed to an RPE plate. We used a line illumination confocal Raman microscope operating at the excitation wavelength of 532 nm.

**Fig. 6:**
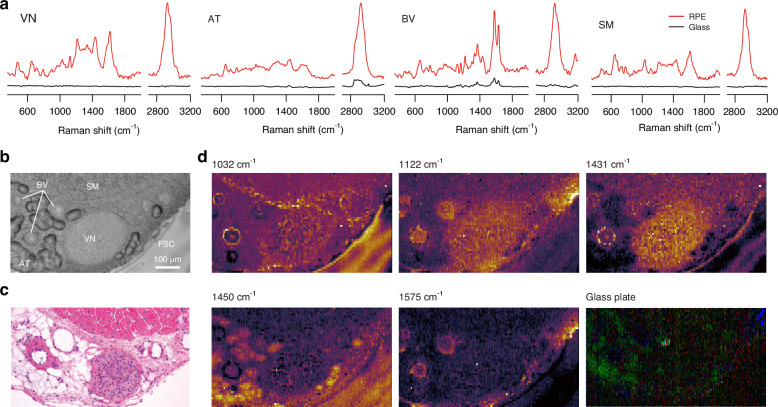
RPE-enhanced Raman spectroscopy of oesophagus with oesophageal adventitia in a Wistar rat. **a** Raman spectra obtained on the RPE plate (red) and the glass plate (black) for different tissue domains. **b** White-light image of the tissue section used for Raman detection. **c** Hematoxylin-eosin-stained image of the serial section. **d** RPE-enhanced Raman images of 5 representative Raman bands within the same region as the white-light image. The excitation wavelength, the objective lens, the excitation power, and the exposure time were 532 nm, ×10 with NA 0.3 and 60 µW/µm^2^, and 2 s/line, respectively. A representative Raman image of a serial section attached to a slide glass plate acquired using the same experimental conditions as the RPE-enhanced Raman imaging is also shown. The intensities of three Raman bands are represented by pseudo-colours: 476 cm^−1^ (blue), 1032 cm^−1^ (red), and 1450 cm^−1^ (green). These Raman bands are selected because they can be reliably measured even with the glass plate when the excitation light intensity and exposure time are sufficient. VN vagus nerve, AT adipose tissue, BV blood vessel, SM smooth muscle, FSC frozen section compound

Raman spectra obtained from different tissue domains are shown in Fig. 6a. The tissue domains were identified using the white-light image of the tissue section for Raman detection (Fig. 6b) and the hematoxylin-eosin-stained images of the serial section (Fig. 6c). By utilising the RPE plate (red spectra), we observed distinct Raman spectra of various components such as vagus nerves, adipose tissues, blood vessels, and smooth muscles. In contrast, when using a slide glass plate (black spectra), Raman spectra were not observable for most tissues. Discernible Raman signals could be obtained from only adipose tissue and blood vessels on the slide glass plate due to the high concentration of fatty acids present in adipose tissue and the occurrence of the resonance Raman effect during the detection of haemoglobin in the blood. Notably, the RPE-enhanced Raman signals to originate from tissues other than fatty acids and haemoglobin exhibited an EF of approximately 10^4^ or higher, as determined by the noise level observed in the Raman spectrum on the glass plate. Conversely, the EFs of RPE-enhanced Raman signals derived from fatty acids and haemoglobin were around 10.

Additionally, we evaluated the tissue imaging capability of RPE, as shown in Fig. 6d. The RPE-enhanced Raman images revealed distinct patterns that effectively highlighted different tissue domains. In contrast, no distinct Raman image was observed using a glass plate, except for blood, which exhibited resonance Raman scattering, and fat, which had a high molecular density. Notably, two images at Raman shifts of 1431 and 1450 cm^−1^ exhibited a sharp transition in contrast from vagus nerves to adipose tissue, despite the minor difference in Raman shift. This finding suggests that RPE-enhanced Raman imaging exhibits a high sensitivity to subtle differences in the Raman shift associated with δ(CH) between the vagus nerve and adipose tissue domains.

## Discussion

The conventional plasmonic enhancement of fluorescence and Raman scattering has been achieved using metal nanostructures that are in close proximity to analyte molecules. However, our study discovers a new possibility for enhancement called RPE, which significantly reduces the range limitation. We have successfully demonstrated RPE using AgNIs with a CSS overlayer that is more than 100 nm thick in fluorescence and Raman spectroscopy. The CSS layer provides physical and chemical protection, reducing the mutual impact between analyte molecules and metal nanostructures. We have confirmed the enhancement effect of RPE with various molecules, including biological samples. The RPE plate also offers sufficient spatial resolution for identifying and distinguishing cells and tissues. Additionally, we have validated the practical advantages of using RPE plates, including improved quantitativeness in molecular detection, production and storage stability, and durability against chemical and physical impact. This novel enhancement modality of fluorescence and Raman spectroscopy provides valuable insights into the mechanism of signal enhancement in photon emission and expands the applications of enhanced spectroscopy.

Our sincere and multifaced demonstrations suggest a certain interaction between the plasmons of AgNIs and the analyte molecules. We conducted electric field calculations based on the finite-difference time-domain method, which indicated an EF of around 5 on the CSS surface, as depicted in Fig. S10. However, this level of enhancement does not correlate with the significant enhancement effects observed in our study, suggesting that the interactions extend beyond the scope of conventional electromagnetic and near-field theories. Therefore, it is necessary to discuss this abnormal enhancement behaviour. Based on our experimental results, one plausible interpretation is that the enhancement can be attributed to remote coupling in resonance between the metal nanoislands and the analyte molecules or dipolar coupling between the giant plasmon dipole of localised surface plasmon resonance and the molecular transition dipole of the analyte molecules. This resonant coupling mechanism can be inferred from several aspects. Firstly, the signal equivalence between the front- and rear-side optical configurations suggests that the enhanced fluorescence and Raman scattering may originate from AgNIs, even though the emission spectra reflect the electronic and vibrational states of the analyte molecules. Secondly, when the plasmonic properties of the AgNIs degrade without affecting the CSS, the enhanced fluorescence and Raman scattering signals weaken, indicating an interaction between the AgNIs and the analyte molecules. Thirdly, the wavelength dependency suggests that the enhancement of fluorescence and Raman scattering relies on the electronic state of the analyte molecules. Importantly, this mechanism implies that plasmon (localised surface plasmon resonance) energy is not transferred to the analyte molecules over the submicrometer distance. Instead, the resonant coupling between the metal nanoislands and the analyte molecules modulates the plasmonic emission spectra via the CSS.

If our hypothesis is correct, the molecular-dependent behaviour of RPE observed in this study can be explained. At higher molecular concentration conditions, the intermolecular distance between analyte molecules becomes shorter, and Förster-type transition dipole-dipole coupling between the analyte molecules may occur more frequently due to resonant excitation with light^44^. Under these conditions, the resonant coupling between the metal nanoislands and the analyte molecules, if present, would be disturbed. This effect may explain the concentration-dependent Raman enhancement observed in the resonant case. At concentrations of 100 μM or higher of FUC, the average intermolecular distance is expected to be 25 nm or less, leading to a frequent random pairing of molecules within the Förster distance of approximately 6 nm. Consequently, the concentration-dependent Raman enhancement may no longer be observed at higher molecular concentrations, possibly due to the disturbance of the resonant coupling between the metal nanoislands and the analyte molecules. However, when the excitation wavelength deviates from the resonance wavelength, where the molecular transition dipole is very small and the intermolecular coupling is insignificant compared to the plasmon-molecule coupling, the Raman enhancement according to molecular concentration is still observed, albeit with a decreased Raman EF. As for PVA, we used it as a polymer matrix to embed analyte molecules. However, we observed no significant Raman signals associated with PVA under any excitation conditions. This observation is consistent with the expected mechanism of RPE since PVA is transparent to visible light wavelengths, resulting in minimal transition dipoles when excited by visible light. Therefore, PVA serves as an RPE-inert polymer matrix for dispersing arbitrary analyte molecules of interest. The resonant coupling between metal nanoislands and analyte molecules is not mediated over long distances in common bulk materials. Thus, CSS likely acts as a structure that mediates long-range resonant coupling between the metal nanoislands and the analyte molecules.

The CSS, divided by the directional grain boundaries, creates a strongly anisotropic solid medium. This anisotropy of CSS plays a crucial role in mediating the long-range resonant coupling. One piece of evidence is the significant enhancement observed in RPE plates with distinguishable intercolumn boundaries, even for CSS thicknesses exceeding 500 nm, whereas a virtually columnless silica structure reduces the enhancement effect, as shown in Fig. 3e. Another piece of evidence is the further enhancement of RPE by the gold(I)/halide bath treatment (Fig. S4). The gold(I)/halide bath treatment likely decorates the intercolumn boundaries of CSS with strongly polarisable halide ions. This modification of the intercolumn boundaries facilitates the long-range resonant coupling between metal nanoislands and analyte molecules. One possible mechanism for mediating the long-range resonant coupling is the directional propagation of polaritons or charge density waves along the CSS columns, which can be modulated based on the presence and state of the CSS interface. However, at present, we lack theoretical proof for this assumption. If this hypothesis is correct, a similar promotion effect of RPE could also be achieved with a gold-free halide bath treatment. In fact, we observed a remarkable increase in RPE activity with plain halide bath treatment (data not shown).

The results of our study suggest that RPE is a promising platform for enhanced biosensing and biomolecular analysis. The most significant advantage of RPE is its ability to substantially enhance fluorescence and Raman signals without the need for close proximity between metal nanostructures and biological specimens. The CSS layer, with a minimum thickness of 100 nm, acts as a robust protective layer, allowing AgNIs to withstand highly corrosive solution environments commonly used in biological experiments, as demonstrated in Fig. 4c. Additionally, the physical robustness, as shown in Fig. 4d, reduces the impact of shear forces on AgNIs caused by sample attachment and media fluid during sample preparation. This feature enables the direct cultivation of live cells on the CSS protection layer of the RPE plate using a culture medium, and thin-sliced biological tissues can be easily attached to the RPE plate. Analyte molecules placed at a distance of approximately 100 nm from the metal surface no longer experience short-range electronic interference from the metal. Consequently, RPE enables rapid and sensitive molecular sensing in a more biocompatible environment.

Furthermore, RPE offers additional benefits. In tissue Raman imaging, RPE may utilise resonant coupling between metal nanostructures and analyte molecules, allowing sensitive reflection of the electronic properties of the respective tissue materials in the Raman EFs. This enables unique Raman imaging that reflects both the vibrational and electronic states of the analyte molecules. In fluorescence biosensing for live cells, the distinctive feature of RPE is its strong preference for low analyte concentrations, which provides an advantage in reducing interference effects caused by external fluorescence probes. For example, an adverse impact of fluo3-AM load may occur at the highest fluo3-AM loading level of 1.0 μM. The fluorescence intensities of fluo3-AM in a substantial portion of HeLa cells reached values higher than 1000 counts, allowing the analysis of Ca^2+^ oscillation in both the RPE plate and slide glass, as shown in Fig. 5. However, the number of oscillations tended to be lower than that observed at lower concentration conditions in both the RPE plate and slide glass. This can be understood in terms of the positive feedback mechanism of cells in regulating intracellular Ca^2+^. In this mechanism, the initial Ca^2+^ release from internal Ca^2+^ stores, corresponding to the first spike, promotes Ca^2+^ influx and subsequent oscillations. Sequestration of free Ca^2+^ by complexing with excess fluo3-AM in the overdosed cells results in an insufficient initial Ca^2+^ rise, impairing the intracellular dynamics of the positive feedback loops. On the other hand, on the RPE plate at low concentration conditions, the weakened fluorescence signals are sufficiently amplified with a lesser adverse effect of fluo3-AM load on Ca^2+^ signalling. This enables more accurate counting of Ca^2+^ oscillations.

In conclusion, we demonstrated RPE by a dense random array of AgNIs with a CSS overlayer of more than 100 nm thickness. The RPE plate presented in this study offers practical advantages for potential biosensing and biomolecular analysis. The fabrication of the RPE plate involves sputtering and chemical immersion processes, which allow for the easy production of large-area RPE plates. The biocompatible structure of the RPE plate, incorporating a CSS layer, creates a more suitable environment for molecular detection, minimising the mutual impact between analyte molecules and metal nanostructures. This feature promises high analytical sensitivity and reduced acquisition time for biosensing and biomolecular analysis without requiring instrumental modifications or specific sample manipulations. While the exact mechanism behind RPE remains unclear and further studies are needed, we anticipate that RPE will emerge as a versatile analytical tool in the fields of chemistry, biology, and medicine. Particularly, it can potentially enhance the biosensing and biomolecular analysis of biological tissues and cells through Raman and fluorescence analysis.

## Materials and methods

### Materials

Most of the chemicals used in this study were of special reagent grade and were obtained from Wako Pure Chemical Industries. PVA with a polymerisation degree of 500 or less and a saponification degree of 86–90% was used. R6G was obtained from Exciton as a chloride salt. PBS was obtained from Sigma-Aldrich. One PBS tablet was dissolved in 200 ml of distilled water, and the pH was adjusted to 7.4. Fluo3-AM, an intracellular Ca^2+^ indicator, was acquired from Dojindo Laboratories. Matrigel, an extracellular matrix, was obtained from Corning. The frozen section compound (FSC 22; Leica Biosystems) used for sample preparation was from Leica Biosystems.

### Preparation of RPE plates

RPE plates were prepared by growing a dense array of AgNIs through direct-current Ar^+^ ion sputtering on the smoother side of a float slide glass plate (Type S7213; Matsunami) in an apparatus similar to that used elsewhere^45^. A glow discharge at a negative voltage of 1.4 kV or less was supplied to the cathode as an Ag target, and a discharge current of 15 mA was used to produce a dense random array of AgNIs within 5 min. The deposition of a CSS layer on the AgNIs layer was conducted using radio-frequency sputtering (model RFS-200; Ulvac) at an Ar pressure of 1.0 Pa. The radio-frequency power was adjusted to 100 W, resulting in a SiO_2_ deposition rate of 10 nm/min. The high-energy discharge plasma caused the substrate temperature to rise up to 160 °C.

### Structural and optical characterizations of RPE plates

The structural and optical characterisations of the RPE plates were carried out using various techniques. FE-SEM (SU5000, SU8000, and SU9000; Hitachi) and energy-dispersive X-ray spectroscopy installed in the FE-SEM (SEM-EDX; SU5000; Hitachi) were used for imaging and elemental analysis. The platinum coating was applied to the samples for FE-SEM imaging to reduce charge accumulation, and osmium coating was used for SEM-EDX measurement. The acceleration voltage for FE-SEM imaging was set at 3–6 kV, while the acceleration voltage for SEM-EDX measurement was set at 6 kV. Spectroscopic ellipsometry (FE-5000; Otsuka Electronics), ultraviolet-visible absorption spectroscopy (UV-3600; Shimadzu), and XPS (ESCA5800; ULVAC-PHI, and ESCA750; Shimadzu) were used for further characterisation.

### Gold(I)/halide bath treatment

The gold(I)/halide solution was prepared as follows: 0.5 g of KSCN was added to 40 mL of a 0.1 wt% solution of NaAuCl_4_·2H_2_O. The mixture was heated to boiling for a few minutes. Then, 0.6 g of NaBr was added. The solution was diluted with water to a total volume of 1 L. Additionally, the solution was diluted with a 0.2 M NaBr solution by a factor of 30. This preparation resulted in a gold(I)/halide bath containing Au(SCN)^2-^ at a concentration of 3.3 μM. To process the RPE plates, the following steps were carried out: The RPE plates were subjected to treatment at 65 °C for 1–2 min. Then, they were rinsed with water and immersed in a 0.2 M NaCl solution for 5 min at 75 °C. Afterwards, the AgNIs were rinsed again with water and dried under ambient air conditions.

### Spectroscopic measurements

A large body of the fluorescence and Raman spectra were measured using a laboratory-made spectrometer comprising a low-power He-Ne laser (633 nm, 0.7 mW) or a green diode laser (532 nm, 3 mW), two lenses in series to collect the emission from the sample on the RPE plate onto an aperture of a light receiver head, connected via fibre optics to the cooled multichannel analyser (PMA-11 and C5966-31; Hamamatsu Photonics).

High-resolution Raman spectra for the excitation laser wavelength of 633 nm were acquired with a commercially available Raman spectrometer (DXR3 SmartRaman Spectrometer; ThermoFisher Scientific). High-resolution Raman spectroscopy and imaging were conducted using a line illumination confocal Raman microscope (Raman-11; Nanophoton) with the excitation laser operating at 532 nm.

Fluorescence images of the cells were captured by an electron-multiplying charge-coupled device camera (ImagEM; Hamamatsu Photonics) operated with MetaFluor software (Molecular Devices). The excitation wavelength was 480 nm. We acquired a time series of fluorescence images of multiple cells captured simultaneously. From these images, the temporal behaviour of the fluorescence intensities of selected cells was extracted.

### Cultivation of cells on RPE plates and incorporation of fluorescent biosensors

HeLa cells obtained from ATCC were routinely cultured with Dulbecco’s modified Eagle medium containing 10% fetal bovine serum, 30 units/mL penicillin, and 30 μg/mL streptomycin under a 95% air, 5% CO_2_ atmosphere at 37 °C. For Ca^2+^ measurement in HeLa cells, 5 × 10^4^ cells were plated onto a Matrigel-coated RPE plate or glass plate. After 2–6 hours, cells were loaded with various concentrations of fluo3-AM at 37 °C for 30 min and washed with Tyrode’s solution (140 mM NaCl, 5 mM KCl, 1 mM MgCl_2_, 2 mM CaCl_2_, 10 mM glucose, and 10 mM HEPES, pH adjusted to 7.4 with NaOH). Histamine was applied to evoke intracellular calcium oscillation. The fluo3-AM fluorescence was measured in Tyrode’s solution at ambient temperature.

### Preparation of biological tissues

All animal experiments were conducted with the approval of and in accordance with guidelines from the Committee for Animal Research, Kyoto Prefectural University of Medicine (Permission No. M25-109). The oesophagus with oesophagal adventitia was excised from a Wistar rat after euthanasia. The oesophagus with oesophagal adventitia was immediately embedded in frozen section compound, snap-frozen in dry ice-acetone, and stored at −80 °C until cryostat sectioning. The frozen samples were sliced into 5 μm thickness using a cryostat microtome (CM1900; Leica) and mounted without any fixation on an RPE plate or a 0.17-mm thickness cover glass (No.1; Matsunami).

## Supplementary information

Supplementary Information

## Data Availability

The data that support the findings of this study are available from the corresponding author upon reasonable request.
