# Supplementary material for: Long-range enhancement for fluorescence and Raman spectroscopy using Ag nanoislands protected with column-structured silica overlayer
Source: Light Sci Appl. 2024 Oct 28;13:299. doi: 10.1038/s41377-024-01655-3 (PMC11514291; doi:10.1038/s41377-024-01655-3)
Supplement: Supplementary file 1 — Supplementary Information [file 41377_2024_1655_MOESM1_ESM.pdf]

Supplementary Information for

Long-range enhancement for fluorescence and Raman spectroscopy using Ag nanoislands protected with column-structured silica overlayer

Takeo Minamikawa,[1,2,3,4,*,§] Reiko Sakaguchi,[5] Yoshinori Harada,[3] Hiroki Tanioka,[2] Sota Inoue,[2] Hideharu Hase,[6] Yasuo Mori,[5,6] Tetsuro Takamatsu,[3,7] Yu Yamasaki,[8] Yukihiro Morimoto,[8,9] Masahiro Kawasaki,[10] and Mitsuo Kawasaki[10,§]

[1]*Department of Systems Innovation, Graduate School of Engineering Science, Osaka University, Osaka 560-8531, Japan.*
[2]*Division of Interdisciplinary Researches for Medicine and Photonics, Institute of Post-LED Photonics, Tokushima University, Tokushima 770-8506, Japan.*
[3]*Department of Pathology and Cell Regulation, Graduate School of Medical Science, Kyoto Prefectural University of Medicine, Kyoto 602-8566, Japan.*
[4]*PRESTO, Japan Science and Technology Agency (JST), Tokushima 770-8506, Japan.*
[5]*Institute for Integrated Cell-Material Sciences (iCeMS), Kyoto University, Kyoto 606-8501, Japan.*
[6]*Department of Synthetic Chemistry and Biological Chemistry, Graduate School of Engineering, Kyoto University, Kyoto 615-8510, Japan.*
[7]*Department of Medical Photonics, Graduate School of Medical Science, Kyoto Prefectural University of Medicine, Kyoto 602-8566, Japan.*
[8]*Technology & Engineering Division, Ushio Inc., Hyogo 671-0224, Japan.*
[9]*The Institute of Science and Industrial Research, Osaka University, Osaka 567-0047, Japan.*
[10]*Department of Molecular Engineering, Graduate School of Engineering, Kyoto University, Kyoto 615-8510, Japan.*
[§]*These authors contributed equally to this work.*
[*]*Corresponding author: minamikawa.takeo.es@osaka-u.ac.jp.*

Table of Contents:

Supplementary Figure S1 ······ **S-2**
Supplementary Figure S2 ······ **S-3**
Supplementary Figure S3 ······ **S-4**
Supplementary Figure S4 ······ **S-4**
Supplementary Figure S5 ······ **S-5**
Supplementary Figure S6 ······ **S-5**
Supplementary Figure S7 ······ **S-6**
Supplementary Figure S8 ······ **S-6**
Supplementary Figure S9 ······ **S-7**
Supplementary Figure S10 ······ **S-7**

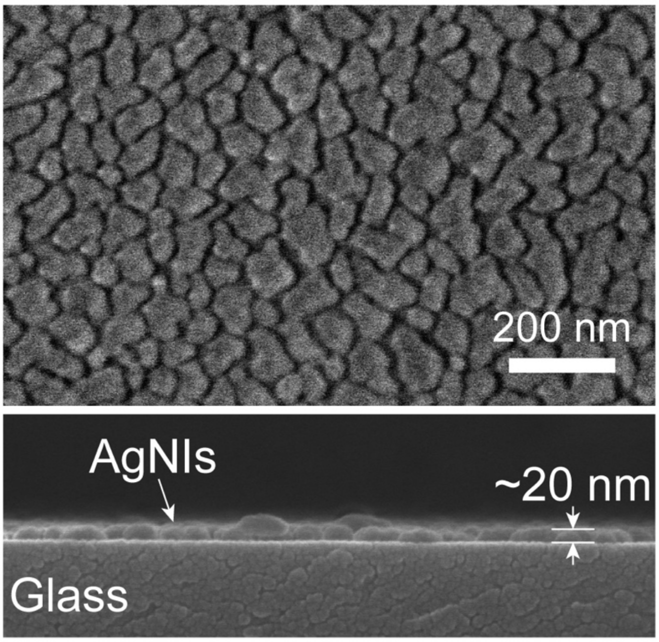

Figure S1. FE-SEM images displaying the top view and oblique cross-section of an AgNI plate.

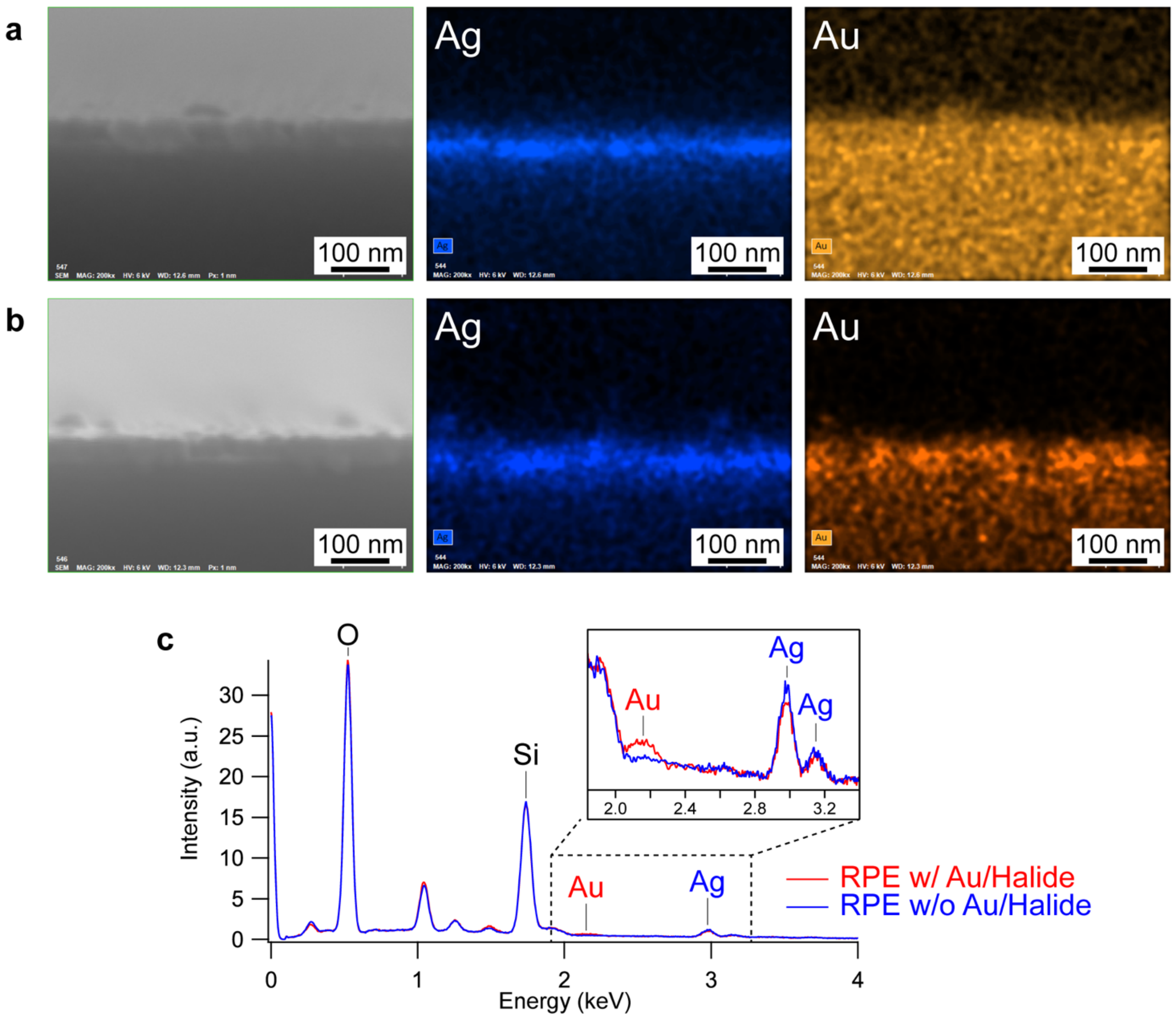

Figure S2. FE-SEM and energy-dispersive X-ray spectroscopy (EDX) analyses of RPE plates. FE-SEM and EDX (Ag and Au) images of RPE plates (a) without and (b) with the gold(I)/halide bath treatment. The morphology of the RPE plate seems not to have changed by the gold(I)/halide bath treatment. The colocalization of Ag and Au was observed after the gold(I)/halide bath treatment. (c) EDX spectra at the AgNIs layer with and without the gold(I)/halide bath treatment.

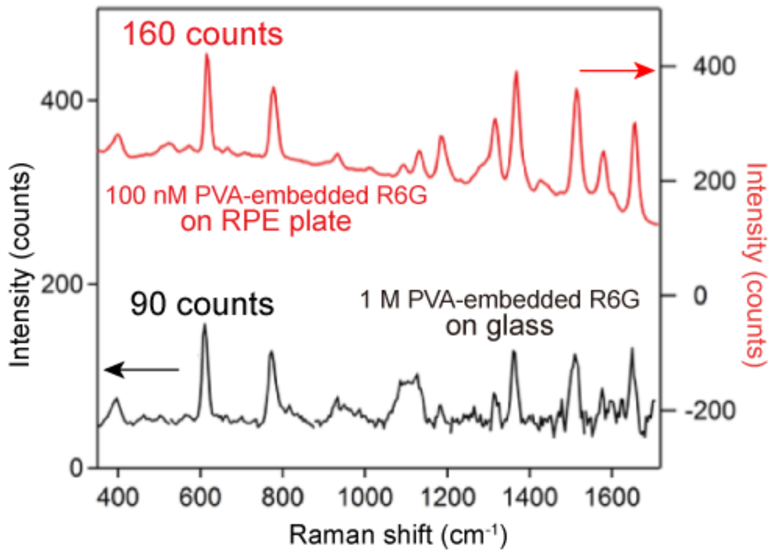

Figure S3. Comparison of 532-nm excited Raman spectra on a slide glass and an RPE plate with the gold(I)/halide bath treatment. Raman spectra of 1 M PVA-embedded R6G dropped on the slide glass and 100 nM PVA-embedded R6G on the RPE plate were observed at the excitation laser power of 3 mW. The thickness of the PVA-embedded 6G in both cases was approximately 4 µm. Assuming that spontaneous Raman scattering is directly proportional to both the excitation laser power and the molecular concentration, a quantitative comparison of the intensities at the 614 cm^{-1} Raman band indicates that the Raman EF provided by RPE is approximately 2×10^7. The reproducibility of EF was evaluated at least 2 times.

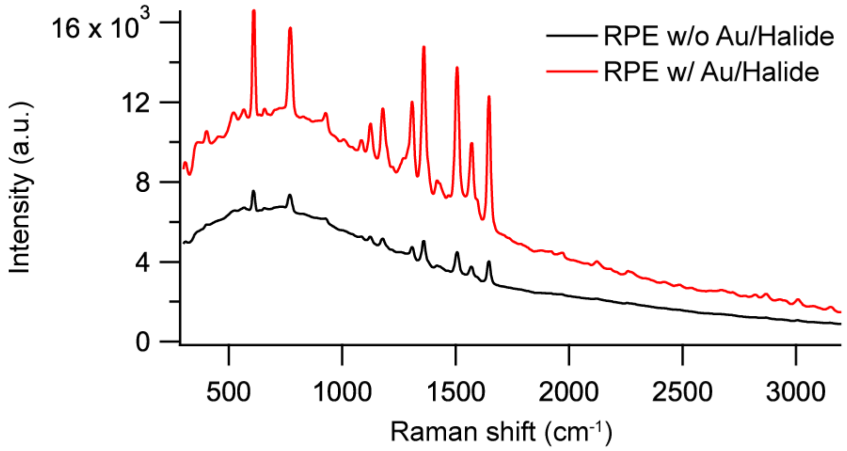

Figure S4. Influence of gold(I)/halide treatment on the RPE-enhanced emission spectrum. R6G molecules, embedded in PVA at a concentration of 100 nM, were deposited onto the RPE plate with and without gold(I)/halide treatment, followed by drying. The emission spectra were measured under an excitation wavelength of 532 nm.

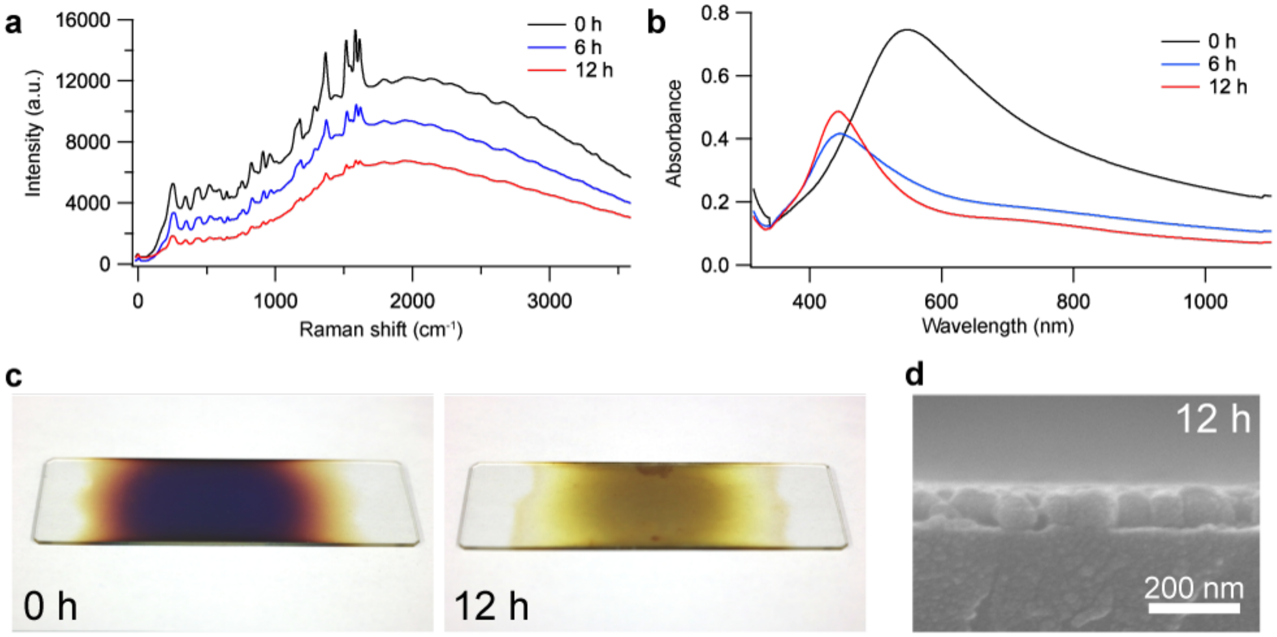

Figure S5. Corrosion effect on RPE plates without the gold(I)/halide treatment by immersing in a 0.16 M NaCl solution at 60ºC. (a) Raman and (b) absorbance spectra of FUC embedded in PVA films on an RPE plate immersed in a NaCl solution. (c) RPE plates before and 12 h after immersion of 0.16 M NaCl solution at 60ºC. The slide glass basal plates measure 26 mm in width and 76 mm in length. (d) A representative FE-SEM image of an RPE plate 12 h after immersion of 0.16 M NaCl solution at 60ºC.

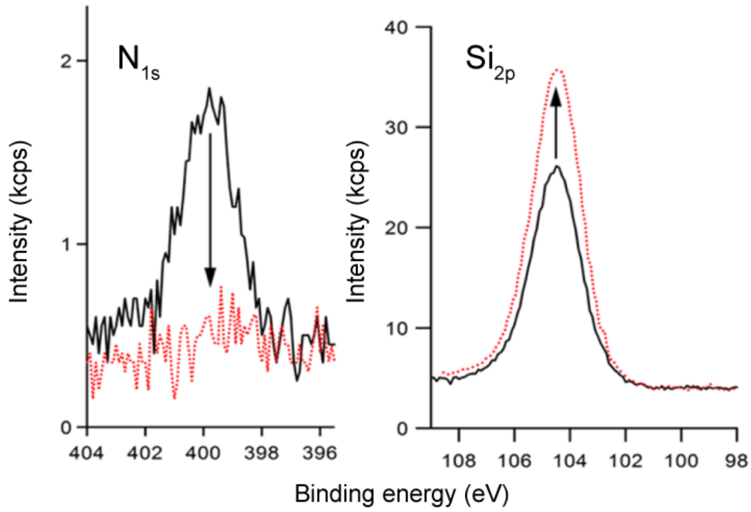

Figure S6. XPS surface analysis of the CSS layer with spin-coated R6G molecules. N_{1s} and Si_{2p} core-level XPS spectra measured for spin-coated R6G a the surface coverage of 3×10^{14} molecules cm^{-2} (solid lines in black) on a CSS layer of 120 nm in thickness over the AgNIs. The sensitivity-corrected N_{1s}/Si_{2p} intensity ratio of ~0.025 is consistent with the confinement of the spin-coated R6G layer within an XPS analysis depth of less than 2 nm. Furthermore, brief ion etching for a nominal depth of less than a few nm was just about enough to eliminate the N_{1s} signal due to R6G (dotted line in red). The resultant cleaner CSS surface without R6Gs caused a noticeably increased Si_{2p} signal associated with the CSS layer (dotted line in red).

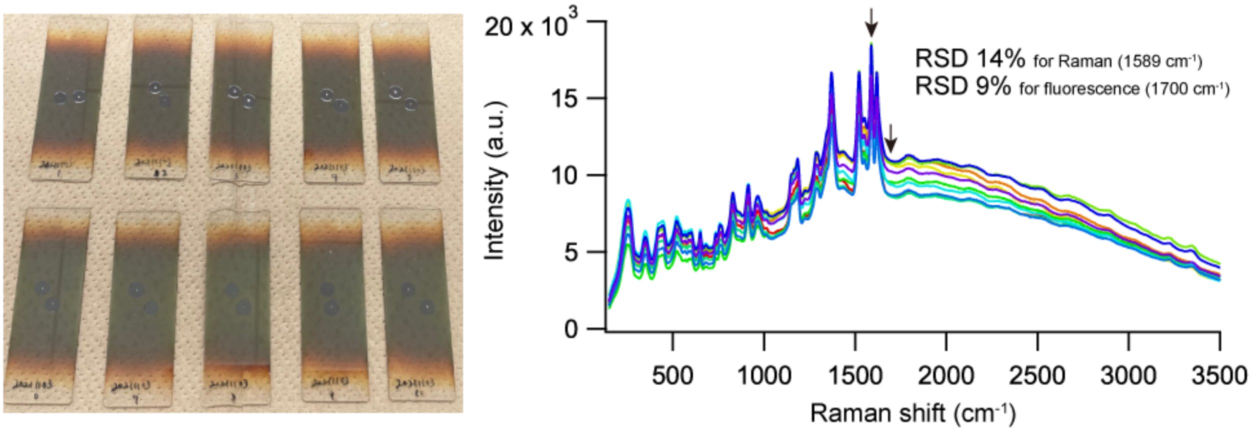

Figure S7. Reproducibility of RPE plates. The Raman spectra of 1 µM PVA-embedded FUC dropped on the RPE plates (N=10). The RSD of Raman intensity at 1589 cm^{-1} of crystal violet was 14%, and that of fluorescence intensity at 1700 cm^{-1} was 9%. The excitation wavelength and power were 532 nm and 0.25 mW, respectively. The slide glass basal plates measure 26 mm in width and 76 mm in length.

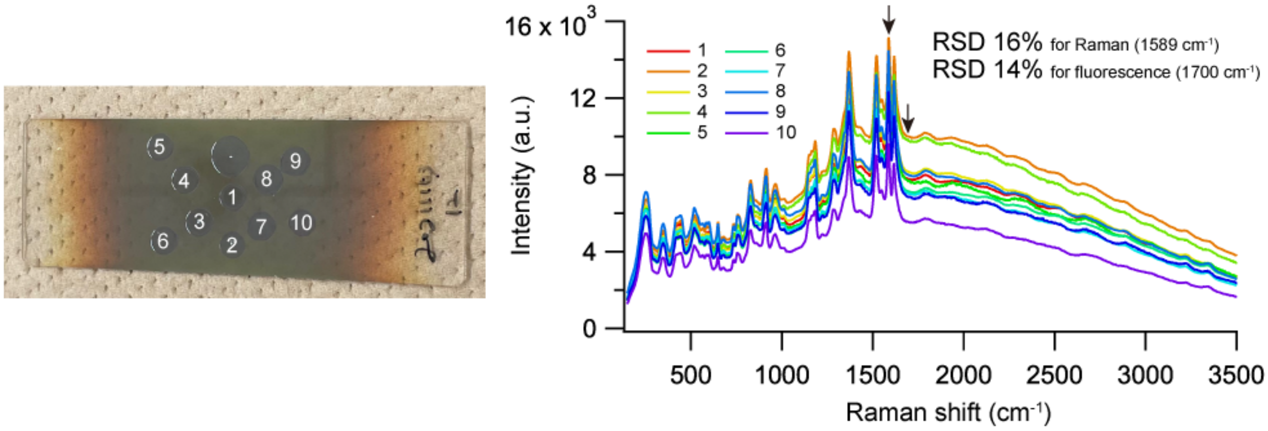

Figure S8. Spatial uniformity of RPE plates. The Raman spectra of 1 µM PVA-embedded FUC dropped on the RPE plates. A total of 10 positions were evaluated. The RSD of Raman intensity at 1589 cm^{-1} of crystal violet was 16%, and that of fluorescence intensity at 1700 cm^{-1} was 14%. The excitation wavelength and power were 532 nm and 0.25 mW, respectively. The slide glass basal plates measure 26 mm in width and 76 mm in length.

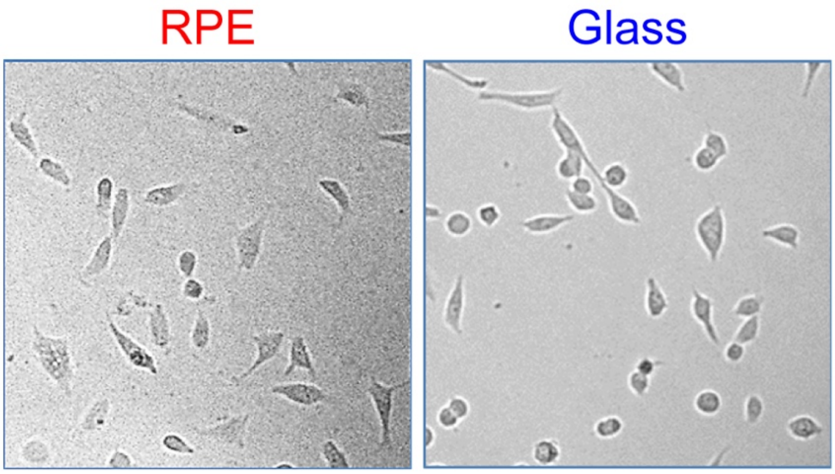

Figure S9. Optical micrographs of HeLa cells cultured on a Matrigel-coated RPE and glass plates.

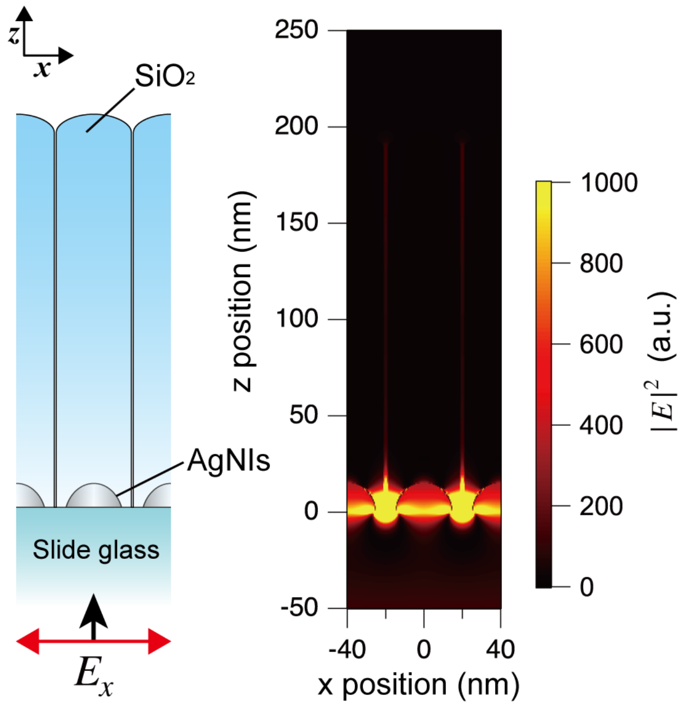

Figure S10. Electric field calculation based on a finite difference time domain method. CSS has a height of 200 nm, a diameter of 38 nm and a gap of 2 nm between CSSs; AgNI has a hemispherical shape and a height of 18 nm. Boundary conditions were periodic boundary in x-direction and perfectly matched layer absorbing boundary in z-direction. An excitation electric field with x polarisation and a wavelength of 532 nm was incident from the slide glass side, and the squared amplitude of the electric field was determined at steady state.
